# Associations of Plasma Phospholipid Omega-6 and Omega-3 Polyunsaturated Fatty Acid Levels and MRI Measures of Cardiovascular Structure and Function: The Multiethnic Study of Atherosclerosis

**DOI:** 10.1155/2011/315134

**Published:** 2011-08-22

**Authors:** Jennifer S. Anderson, Jennifer A. Nettleton, W. Gregory Hundley, Michael Y. Tsai, Lyn M. Steffen, Rozenn N. Lemaitre, David Siscovick, João Lima, Martin R. Prince, David Herrington

**Affiliations:** ^1^Division of Cardiology, Deparment of Internal Medicine, Wake Forest University, Winston-Salem, NC 27157, USA; ^2^Division of Epidemiology, Human Genetics and Environmental Sciences, University of Texas Health Sciences Center at Houston, Houston, TX 77030, USA; ^3^Division of Epidemiology and Community Health, University of Minnesota, Minneapolis, MN 55455, USA; ^4^Departments of Medicine and Epidemiology, University of Washington, Seattle, WA 98105, USA; ^5^Division of Cardiology, Department of Medicine, Johns Hopkins University, Baltimore, MD 2187-0020, USA; ^6^Department of Radiology, Columbia College of Physicians and Surgeons, New York, NY 10032, USA

## Abstract

*Background*. The association between plasma omega-6 fatty acids and cardiovascular disease (CVD) is unclear, and discrepancy remains concerning the cardiovascular benefit of the omega-3 fatty acid alpha-linolenic acid. *Methods*. Associations of plasma phospholipid fatty acid levels (arachidonic acid, linoleic acid, eicosapentaenoic acid, docosahexaenoic acid (DHA), and alpha-linolenic acid) with cardiac magnetic resonance imaging measures of left ventricular (LV) mass, LV volume, ejection fraction, stroke volume, and aortic distensibility were investigated in 1,274 adults. *Results*. Results of multivariate analysis showed no statistically significant associations of plasma omega-6 or omega-3 levels with cardiac magnetic resonance imaging measures. Stratification by gender revealed a positive association between DHA and LV mass in women (*β* = 1.89, *P* = 0.02; *P* interaction = 0.003) and a trend for a positive association between DHA and ejection fraction in men (*β* = 0.009, *P* = 0.05; *P* interaction = 0.03). *Conclusion*. Additional research is warranted to clarify the effects of plasma DHA on cardiac structure and function in women versus men.

## 1. Introduction

Contemporary work suggests that consumption of polyunsaturated fatty acids (PUFAs), in place of saturated fats, decreases the risk of cardiovascular disease (CVD) [1]. In addition, dietary long-chain omega-3 PUFAs (eicosapentaenoic acid (EPA) and docosahexaenoic acid (DHA), both found in fatty fish) have been associated with measurable improvements in cardiac hemodynamics and function as assessed by echocardiography. However, more accurate objective measures assessing the relationship of PUFAs and LV mass as well as LV mass/volume ratio (the latter associated with both nonheart failure cardiovascular events and diastolic dysfunction [2]) in human subjects are lacking. Furthermore, similar associations among fatty acids other than long-chain omega-3 PUFAs have not been defined. Thus, the purpose of this study is to determine the associations between plasma phospholipid omega-6 PUFAs (arachidonic acid (AA) and linoleic acid (LA)) and plasma phospholipid omega-3 PUFAs (EPA, DHA, and ALA) with cardiac magnetic resonance (CMR) measures of cardiovascular structure and function, including aortic distensibility, LV mass, LV mass/volume ratio, ejection fraction, and stroke volume.

## 2. Materials and Methods

### 2.1. Study Population and Data Collection

MESA is a prospective cohort study that began in July 2000 to investigate the prevalence, correlates, and progression of subclinical CVD in individuals without known CVD at baseline [3]. The main cohort included 6,814 women and men aged 45–84 years old at baseline recruited from 6 US communities. MESA cohort participants were 38% white (*n* = 2624), 28% black (*n* = 1895), 22% Hispanic (*n* = 1492), and 12% Chinese (*n* = 803). A variety of noninvasive measures of subclinical disease, including magnetic resonance imaging of cardiac structure and function, were obtained from volunteers during the first examination of the MESA cohort (July 2000–August 2002). In the present study, we excluded those who had missing data on both plasma phospholipid fatty acids (*n* = 5,172) and CMR measures (*n* = 4,404 for measures of LV mass, LV mass/volume ratio, ejection fraction, and stroke volume; *n* = 5,136 for aortic distensibility) as well as those with missing covariates used in the study (*n* = 2). Because plasma phospholipid fatty acids and CMR measures were secondary measures, these assessments were not performed on the full cohort at baseline. Total sample size of participants who had data on plasma phospholipid measures, selected covariates, and CMR measures of LV mass, LV mass/volume ratio, ejection fraction, and stroke volume was 1,274. Total sample size of subjects who had data on plasma phospholipid measures, selected covariates, and CMR measures of aortic stiffness was 914. This study was approved by the Institutional Review Boards of each study site, and written informed consent was obtained from all participants.

### 2.2. Plasma Phospholipids Extraction

Fasting blood samples were collected, and fatty acid analyses were performed at the Collaborative Studies Clinical Laboratory at Fairview-University Medical Center (Minneapolis, Minn, USA) as previously described [3, 4]. In brief, plasma phospholipids were extracted with chloroform/methanol, and thin layer chromatography was used to separate the lipid fractions. The fatty acids in the phospholipid fraction were transmethylated and separated by gas chromatography equipped with a flame ionization detector. The concentration of each fatty acid was expressed as a percentage of total fatty acids. For statistical analyses, plasma phospholipid fatty acids were divided into quartiles. 

### 2.3. Cardiac Magnetic Resonance Imaging Measures

CMR imaging was performed with 1.5-T whole-body MRI systems, Signa CV/I or Signa LX (General Electric Medical Systems), at the first examination. Determination of LV mass and volumes have been previously described and were adjusted for body size [5]. LV mass was determined by the summation of myocardial area (the difference between endocardial and epicardial contour) times slice thickness plus image gap in the end-diastolic phase multiplied by the specific gravity of myocardium (1.05 g/mL). LV end-diastolic volume and LV end-systolic volume were calculated by the summation of areas on each separate slice multiplied by the summation of slice thickness and image gap. LV stroke volume was calculated as the difference between LV end-diastolic volume and LV end-systolic volume. LV ejection fraction was calculated as LV stroke volume divided by LV end-diastolic volume multiplied by 100. 

Evaluation of aortic distensibility has also been previously described [6]. Images of the ascending and descending aorta were acquired in the transverse plane at the level of the right pulmonary artery perpendicular to the vessel lumen. The following formula was used for calculation of aortic distensibility: aortic distensibility = (maximum area − minimum area)/[(Minimum area) × pulse pressure] × 1000. The minimum and maximum cross-sectional areas of the ascending aorta were determined using an automated contour routine using the software FLOW (Medis). Pulse pressure was the difference between systolic and diastolic measurements of blood pressure, obtained immediately before and after the MRI aortic measurements while the patient was in the supine position in the MRI scanner.

### 2.4. Statistical Analysis

Means and standard deviations or proportions were calculated for selected variables according to quartiles of plasma fatty acids (Table 1). For the primary analysis, linear regression models were used to examine the association between plasma phospholipid fatty acid levels and CMR measures of cardiovascular structure and function. Minimally adjusted (age, gender, race/ethnicity, and field center) and fully adjusted models were used to examine these relationships. A number of potential confounders for inclusion in the fully adjusted models were evaluated based on clinical relevance, previously published associations, or associations with exposures or outcomes in the current data set. For parsimony in model construction, covariates that did not appreciably alter the relationship between plasma fatty acids and CMR measures of cardiovascular structure and function were excluded. The final multivariable adjusted model included age, gender, race/ethnicity, BMI, current smoking status, education level, field center, and total : HDL cholesterol, systolic blood pressure, and total energy intake.

As our previous work has suggested a gender difference in the association between omega-3 fatty acids and endothelial function [7] and because gender differences in fatty acid metabolism and tissue levels have been described [8–11], we also chose to stratify analyses by gender. When there appeared to be a significant difference in plasma phospholipid fatty acid levels and CMR measures, formal tests for interaction were performed using the product of gender × plasma phospholipid fatty acid in the model. Because fatty acids have also been shown to have differential effects on vascular reactivity by age [12], we also explored whether there were significant differences in the relationship between plasma phospholipid fatty acid levels and CMR measures after stratification by age (<65 years versus ≥65 years). All *P* values were 2-sided, and because multiple exposure-outcome tests were performed, a more stringent *P* value of < 0.01 was considered statistically significant. We used JMP version 8.0 (SAS Institute Inc., Cary, NC, USA) for analyses.

## 3. Results

### 3.1. Participant Characteristics

Characteristics of participants according to plasma phospholipid omega-6 fatty acid LA and omega-3 fatty acid DHA quartiles are presented in Table 1. The highest plasma phospholipid LA quartile was associated with male gender, Chinese race/ethnicity, lower BMI, lower blood lipid levels, lower prevalence of diabetes diagnosis, lower alcohol intake, and less fruit intake compared to the lower plasma phospholipid LA quartiles. In comparison, the highest plasma phospholipid DHA quartile was associated with older age, female gender, Chinese race/ethnicity, lower BMI, higher education, less tobacco use, lower systolic blood pressure, lower blood lipid levels, higher cholesterol medication use, lower total energy intake, lower alcohol intake, less saturated fat intake, and greater cruciferous vegetable intake compared to the lower plasma phospholipid DHA quartiles. 

### 3.2. Plasma Omega-6 Fatty Acids and CMR Measures of Cardiovascular Structure and Function

After adjustment for age, gender, race/ethnicity, and site, plasma phospholipid AA showed no association with LV mass, LV mass/volume ratio, EF, stroke volume, or aortic distensibility as assessed by CMR. These results were unchanged after adjustment for additional covariates (Table 2). Plasma phospholipid LA showed a statistically significant inverse association with ejection fraction in the minimally adjusted model (*P* = 0.001) with a trend for significance in the fully adjusted model (*P* = 0.02; Table 2), but this association was not robust after additional adjustment for plasma phospholipid saturated fatty acid levels (*P* = 0.07, data not shown). No other significant associations between plasma phospholipid LA and CMR measures of cardiovascular structure and function were found in either minimally or fully adjusted models (Table 2). Stratification by age and gender revealed no heterogeneity in the relationship between either plasma phospholipid AA or LA and CMR measures of cardiovascular structure and function (data not shown). 

### 3.3. Plasma Omega-3 Fatty Acids and CMR Measures of Cardiovascular Structure and Function

After adjustment for age, gender, race/ethnicity, and site, plasma phospholipid DHA showed no significant association with LV mass, LV mass/volume ratio, EF, stroke volume, or aortic distensibility in either minimally or fully adjusted models (Table 3(a)). Though trends for positive associations between plasma phospholipid EPA and LV mass as well as stroke volume were noted in minimally adjusted models (*P* = 0.02 and *P* = 0.03, resp., data not shown), these associations became insignificant after adjustment for additional covariates (Table 3(a)). Similarly, plasma phospholipid ALA showed no statistically significant associations with LV mass, LV mass/volume ratio, EF, stroke volume, or aortic distensibility as assessed by CMR. These associations were unchanged after adjustment for additional covariates (Table 3(b)), including adjustment for other plasma fatty acids. 

**Figure 1:**
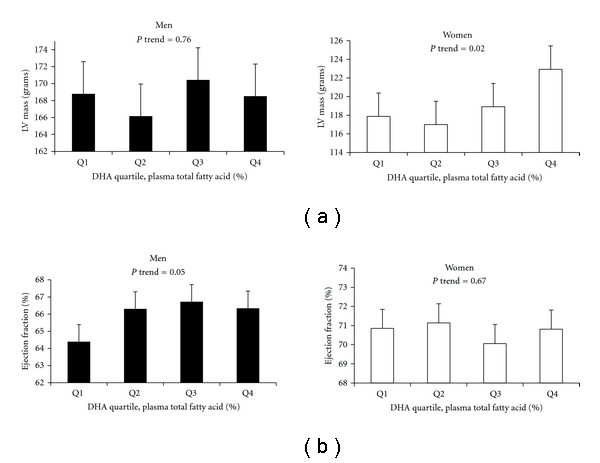
Relations between plasma phospholipid docosahexaenoic acid (DHA) levels, left ventricular (LV) mass, and ejection fraction, by gender. Results shown are least squares means ± SD after adjustment for age, race/ethnicity, BMI, current smoking status, education level, site, total : HDL cholesterol, systolic blood pressure, and total energy intake. Concentrations of plasma phospholipid fatty acid levels (expressed as percentages of total fatty acids) including DHA were summed and ranked into quartiles from lowest to highest based on sample range. Significant gender × DHA interactions were noted for both LV mass (*P* = 0.003) and ejection fraction (*P* = 0.03).

When analyses were stratified by gender, a trend for a positive association was noted between plasma phospholipid DHA and LV mass in women (*P* = 0.02; Figure 1(a)) but not in men, and a statistically significant gender × DHA interaction was observed (*P* = 0.003; mean plasma phospholipid fatty acid levels for each quartile by gender are shown in Table 4). A similar pattern between plasma phospholipid EPA and LV mass in women was observed, but again, results were not statistically significant (*P* = 0.06, fully-adjusted model); tests for gender × EPA interactions were also nonsignificant. In contrast to the findings noted in women, higher plasma phospholipid DHA was associated with a trend for increased ejection fraction in men (*P* = 0.05; Figure 1(b)); formal gender × DHA interaction tests also showed a trend toward significance (*P* = 0.03). Finally, stratification by age revealed no heterogeneity in the relationship between plasma phospholipid omega-3 fatty acid levels and CMR measures of cardiovascular structure and function (data not shown). 

## 4. Discussion

Plasma phospholipid polyunsaturated fatty acids are a marker of dietary patterns, with higher proportions generally reflecting greater consumption of polyunsaturated fats and less cardiovascular disease. These data from 1,274 healthy, multiethnic volunteers corroborate this concept by showing that men within higher plasma phospholipid DHA quartiles (with quartiles two through four corresponding to average intake of one or more servings of nonfried fish per week, as compared with food frequency questionnaire data and as supported by others [13, 14]) had modestly higher ejection fractions compared to men within lower plasma phospholipid DHA quartiles (quartile one corresponding to average intake of less than one serving of nonfried fish per week.) In contrast, women within higher plasma phospholipid DHA quartiles demonstrated modestly higher LV mass compared to women within lower plasma phospholipid DHA quartiles. Though MRI measures of cardiac structure and function are considered highly accurate and reproducible, the clinical significance of such small differences among a healthy population remains unclear. Lastly, the present analysis does not provide evidence of any significant associations between plasma phospholipid omega-6 or ALA PUFAs and CMR measures of cardiovascular structure and function. 

The beneficial effects of dietary EPA and DHA in prevention of sudden cardiac death, acute coronary syndrome, and heart failure are well documented [15–19]. Consumption of long-chain omega-3 fatty acids has been associated with improvements in stroke volume, systemic vascular resistance, and improved E/A ratio (a marker for diastolic dysfunction) as assessed by echocardiography [20], and an inverse association between plasma DHA and measures of arterial stiffness including pulse wave velocity has been observed [21]. These findings are biologically plausible, as experimental evidence suggest that long-chain fatty acids may impact membrane fluidity, nitric oxide production, and/or shift of eicosanoid pathways involved in inflammation and vasoconstriction [22–25]. 

In contrast to the results in men, the observed positive association between plasma phospholipid DHA and increased LV mass among women within in this cohort initially appears surprising. However, there remains considerable discrepancy in the literature regarding gender differences in the association between PUFAs and cardiovascular outcomes. Among population-based cohorts, the nurses' health study demonstrated a significantly negative association between fish consumption and incidence of CHD, and the ARIC study showed an inverse relationship in plasma phospholipid omega-3 fatty acids (particularly DHA) and incident heart failure in women [26]. Conversely, the Rotterdam study observed no relationship between fish consumption and incident heart failure in a population of predominantly women [27]. Other studies in women have observed an inverse relationship between PUFA intake and HDL cholesterol concentrations [28], evidence of atherosclerotic progression [29], and nonsignificant trends toward more CHD events and higher total mortality compared to men [30]. In a previous report, we found that women within the highest quartile of nonfried fish consumption had decreased brachial artery flow-mediated dilation (FMD), a noninvasive measure of endothelial function, compared to women within the lowest quartile of nonfried fish consumption [7]. 

Endothelial dysfunction is an independent predictor of cardiovascular events [31, 32], and may induce an increased in LV mass via increased systemic vascular resistance [33]. In addition, the effects of estrogen (specifically, 17beta-estradiol) on LV mass have been well established and potentially include several mediators that may function via multiple receptors [34–37]. One such mediator may include the transcription factor sterol regulatory element-binding protein-1 (SREBP-1) [38]. DHA is known to downregulate production of SREBP-1, and others have shown that SREBP-1 is involved in conversion of estrone to 17beta-estradiol [39]. Thus perhaps elevated DHA levels, resulting in downregulation of SREBP-1 with subsequent decreased bioconversion toward 17beta-estradiol, may result in adverse effects on vascular reactivity and/or LV remodeling. Future investigations including animal studies and/or in vitro models may provide additional insight to the validity of this hypothesis.

The strengths of this study include the ethnically diverse population, the inclusion of both men and women, and the availability of objective plasma phospholipid fatty acid measures as well as CMR measures of cardiovascular structure and function. The limitations include the observational cross-sectional study design with its known inability to infer causation and potential for temporal bias, and like any observational study, there remains the possibility of residual confounding for factors (such as health status) that could have meaningful impact on the observed results. It is also recognized that plasma phospholipid fatty acid measures are reflective of dietary intake over weeks, hence may not be representative of long-term intake [40]. Lastly, though we chose a more conservative *a priori P* value in an attempt to correct for any findings that may be reflective of spurious associations resulting from multiple exposure-outcome testing, the vast majority of our observations exceeded this threshold, and must be interpreted with caution. 

In conclusion, this study is the first to examine the relationship between plasma phospholipid fatty acid levels and CMR measures of cardiovascular structure and function. Within this cohort of mixed ethnic/racial groups without known coronary artery disease, a gender difference was suggested in the association between plasma phospholipid DHA and ejection fraction: a positive association between DHA and ejection fraction was observed in men but not women, and a trend for a gender × DHA interaction was noted. In addition, a positive association between plasma phospholipid DHA and LV mass was found among women but not men, and a statistically significant gender × DHA interaction was observed. Additional research is warranted to clarify the effects of omega-3 fatty acids on cardiac structure and function in women versus men. 

## Figures and Tables

**Table 1:** Relation between baseline characteristics and plasma phospholipid DHA, LA.^‡^

	LA quartile (*n*)					DHA quartile (*n*)				
	Q1	Q2	Q3	Q4	*P*	Q1	Q2	Q3	Q4	*P*
	*n* = 324	*n* = 316	*n* = 318	*n* = 318	trend	*n* = 321	*n* = 318	*n* = 321	*n* = 316	trend

Age in years, mean ± SE	65.4 ± 0.7	63.7 ± 0.7	63.7 ± 0.7	62.96 ± 0.8	0.002	64.0 ± 0.9	62.7 ± 1.0	62.5 ± 1.0	62.1 ± 0.7	0.01
Gender, %female	65.5	61.1	61.3	51.8	0.0006	54.2	61.6	60	69.1	0.0008
Race/ethnicity, %										
White	20.2	12.9	14.6	11.1	<.0001	24.3	15.9	4.9	5.1	0.56
Black	22.9	16.5	11.3	4.7		8.2	16.8	19.2	15.9	
Chinese	12.7	19.4	24.2	34.7		4.9	18	37.2	45.8	
Hispanic	44.2	51.1	49.8	49.4		62.7	49.4	38.7	33.1	
BMI, mean ± SE	28.1 ± 0.3	28.4 ± 0.3	27.7 ± 5	27.7 ± 0.3	0.04	28.4 ± 0.3	28.3 ± 0.3	27.7 ± 0.3	27.5 ± 0.3	0.005
Education ≥ high school, %	82	79.5	79.7	83.9	0.51	79.9	81.1	82.8	81.1	0.64
Cigarette smoking, %										
Current	15.1	16.5	20.3	18.7	0.06	21.8	17.6	17.4	11.9	0.0005
Phys activity, mod-heavy										
Mean ± SE MET-h/wk	5143 ± 374	5343 ± 362	5547 ± 366	5446 ± 389	0.35	6193 ± 377	4991 ± 365	4673 ± 378	5386 ± 387	0.08
SBP, mean ± SE mmHg	128 ± 1.4	126 ± 1.3	125 ± 1.3	125 ± 1.4	0.02	127 ± 1.4	124 ± 1.3	127 ± 1.4	126 ± 1.4	0.83
Total : HDL cholesterol	4.3 ± 0.08	4.3 ± 0.08	4.2 ± 0.08	4.2 ± 0.09	0.16	4.3 ± 0.09	4.3 ± 0.08	4.2 ± 0.09	4.2 ± 0.09	0.31
Cholesterol medication, %	25.7	15	9.6	5.9	<.0001	9.1	14.4	18.9	18.2	0.001
Diabetic, %	15.5	17	15.6	15.5	0.43	15.5	17	15.6	15.6	0.86
Total energy, mean										
kcal/day	1591 ± 53	1616 ± 52	1640 ± 52	1592 ± 56	0.87	1797 ± 54	1539±52	1546 ± 54	1512 ± 55	<.0001
Alcohol, avg drinks/wk	4.6	3.7	3.3	2.8	0.02	3.1	3.7	4	3.9	0.35
Saturated fat, %kcal	18.9	20.3	20.5	20.3	0.09	11.2	10.8	10.4	9.8	<.0001
Fruits, avg srv/day	1.7	1.5	1.5	1.6	0.24	1.6	1.5	1.6	1.8	0.05
Cruciferous vegetables, savg srv/day	0.35	0.37	0.4	0.37	0.35	0.33	0.37	0.38	0.44	0.002

^‡^Adjusted for age, gender, race/ethnicity, education, and site.

**Table 2:** Relation between plasma phospholipid omega-6 fatty acid levels and measures of cardiac structure and function.^‡,∗^

Measure	Plasma AA (*n*)^§^					Plasma LA (*n*)^§^				
	Q1	Q2	Q3	Q4	*P*	Q1	Q2	Q3	Q4	*P*
	*n* = 319	*n* = 319	*n* = 320	*n* = 318	trend	*n* = 324	*n* = 316	*n* = 318	*n* = 318	trend
Aortic distensibility, 10(−3) mmHg(−1)	1.25	1.25	1.22	1.23	0.4	1.23	1.25	1.24	1.23	0.98
LV mass, grams	143.3	144.4	145.8	143.8	0.62	144.2	144.4	145.7	142.8	0.69
LV mass : volume	0.12	0.11	0.11	0.14	0.64	0.13	0.13	0.12	0.12	0.70
Ejection fraction, %	68.32	68.05	68.01	68.61	0.67	68.99	68.66	67.52	68.01	0.02
Stroke volume, mL	84.92	86.10	86.82	83.86	0.64	86.30	85.32	85.57	84.27	0.20

^‡^Multivariate linear regression analysis with adjustment for age, gender, race/ethnicity, BMI, current smoking status, education level, site, and total : HDL cholesterol, systolic blood pressure, and total energy intake.

*AA, arachidonic acid; LA, linoleic acid; LV, left ventricular.

^§^Sample sizes are for each plasma phospholipid quartile associated with LV mass, LV mass/volume ratio, ejection fraction, and stroke volume.

Sample sizes for each plasma phospholipid quartile associated with aortic distensibility varied slightly due to fewer available aortic distensibility measures and were as follows for AA: Q1 = 204, Q2 = 232, Q3 = 140, Q4 = 238 and for LA: Q1 = 255, Q2 = 227, Q3 = 223, Q4 = 209.

**Table:** (a)

Measure	Plasma EPA (*n*)^§^				Plasma DHA (*n*)^§^					
	Q1	Q2	Q3	Q4	*P*	Q1	Q2	Q3	Q4	*P*
	*n* = 305	*n* = 298	*n* = 334	*n* = 337	trend	*n* = 292	*n *= 309	*n* = 327	*n* = 346	trend
Aortic distensibility, 10(−3) mmHg(−1)	1.22	1.26	1.23	1.26	0.26	1.23	1.22	1.26	1.26	0.17
LV mass, grams	143.14	143.61	146.48	145.38	0.14	144.14	142.19	145.45	147.19	0.07
LV mass : volume	0.12	0.12	0.14	0.12	0.31	0.12	0.12	0.13	0.14	0.21
Ejection fraction, %	67.94	68.50	68.22	68.68	0.26	68.13	68.82	68.35	68.53	0.29
Stroke volume, mL	84.52	85.93	85.15	87.04	0.12	84.67	85.56	85.47	86.55	0.27

^‡^Multivariate linear regression analysis with adjustment for age, gender, race/ethnicity, BMI, current smoking status, education level, site, and total:HDL cholesterol, systolic blood pressure, and total energy intake.

*EPA, eicosapentaenoic acid; DHA, docosahexaenoic acid; ALA, alpha-linolenic acid; LV, left ventricular.

^§^Sample sizes are for each plasma phospholipid quartile associated with LV mass, LV mass/volume ratio, ejection fraction, and stroke volume.

Sample sizes for each plasma phospholipid quartile associated with aortic distensibility varied slightly due to fewer available aortic distensibility

measures and were as follows for EPA, Q1 = 225, Q2 = 212, Q3 = 245, Q4 = 232 and for DHA, QQ = 213, Q2 = 239, Q3 = 220, Q4 = 242.

**Table:** (b)

Measure	Plasma ALA (*n*)^§^				
	Q1	Q2	Q3	Q4	*P*
	*n* = 345	*n* = 318	*n* = 308	*n* = 303	Trend

Aortic distensibility, 10(−3) mmHg(−1)	1.23	1.26	1.21	1.24	0.59
LV mass, grams	143.41	144.87	145.19	144.67	0.48
LV mass : volume	0.13	0.13	0.11	0.12	0.40
Ejection fraction, %	68.18	68.53	68.20	68.07	0.73
Stroke volume, mL	84.20	85.40	87.46	85.37	0.18

^‡^Multivariate linear regression analysis with adjustment for age, gender, race/ethnicity, BMI, current smoking status, education level, site, and total:HDL cholesterol, systolicblood pressure, and total energy intake.

*ALA, alpha-linolenic acid; LV, left ventricular.

^§^Sample sizes are for each plasma phospholipid quartile associated with LV mass, LV mass/volume ratio, ejection fraction, and stroke volume. Sample sizes for each ALA quartile associated with aortic distensibility varied slightly due to fewer available aortic distensibility measures and were as follows: Q1 = 258, Q2 = 226, Q3 = 199, Q4 = 231.

**Table 4:** Mean plasma phospholipid fatty acid quartile values, by gender. ^‡^

Plasma fatty acid	Men				Women			
	Q1	Q2	Q3	Q4	Q1	Q2	Q3	Q4
AA	8.95 ± 0.18	10.70 ± 0.07	12.39 ± 0.07	15.21 ± 0.20	8.99 ± 0.17	10.61 ± 0.07	12.28 ± 0.07	15.18 ± 0.20
LA	17.46 ± 0.24	20.65 ± 0.10	22.74 ± 0.09	25.59 ± 0.31	17.37 ± 0.24	20.63 ± 0.09	22.70 ± 0.09	25.21 ± 0.29
EPA	0.4 ± 0.01	0.62 ± 0.01	0.95 ± 0.02	2.02 ± 0.25	0.41 ± 0.01	0.63 ± 0.01	0.93 ± 0.02	2.03 ± 0.25
DHA	2.54 ± 0.06	3.69 ± 0.04	4.76 ± 0.05	6.06 ± 0.22	2.64 ± 0.06	3.67 ± 0.04	4.77 ± 0.05	6.4 ± 0.22
ALA	0.10 ± 0.01	0.15 ± 0.01	0.19 ± 0.01	0.29 ± 0.02	0.10 ± 0.01	0.15 ± 0.01	0.19 ± 0.01	0.27 ± 0.02

^‡^Mean ± SE, expressed as %total plasma phospholipid fatty acids and adjusted for age, race/ethnicity, BMI, current smoking status, education level, site, total:HDL cholesterol, systolic blood pressure, and total energy intake.

*AA, arachidonic acid; LA, linoleic acid; EPA, eicosapentaenoic acid; DHA, docosahexaenoic acid; ALA, alpha-linolenic acid.
